# Individual, household, and community-level predictors of modern contraceptive use among married women in Cameroon: a multilevel analysis

**DOI:** 10.1093/inthealth/ihab092

**Published:** 2022-01-12

**Authors:** Betregiorgis Zegeye, Dina Idriss-Wheeler, Bright Opoku Ahinkorah, Edward Kwabena Ameyaw, Abdul-Aziz Seidu, Mpho Keetile, Sanni Yaya

**Affiliations:** HaSET Maternal and Child Health Research Program, Addis Ababa, Ethiopia; Interdisciplinary School of Health Sciences, University of Ottawa, Ottawa, ON K1N 6N5, Canada; School of Public Health, Faculty of Health, University of Technology Sydney, Harris St, Ultimo, NSW 2007, Australia; School of Public Health, Faculty of Health, University of Technology Sydney, Harris St, Ultimo, NSW 2007, Australia; Department of Population and Health, University of Cape Coast, Cape Coast, Ghana; Population Studies and Demography, University of Botswana, Private Bag UB 0022 Gaborone, Botswana; School of International Development and Global Studies, University of Ottawa, Ottawa, ON K1N 6N5, Canada

**Keywords:** Cameroon, DHS, global health, modern contraceptive, multilevel, predictors, women's health

## Abstract

**Background:**

Unintended pregnancy remains a major public health and socio-economic problem in sub-Saharan African countries, including Cameroon. Modern contraceptive use can avert unintended pregnancy and its related problems. In Cameroon, the prevalence of modern contraceptive use is low. Therefore, this study investigated the individual/household and community-level predictors for modern contraceptive use among married women in Cameroon.

**Methods:**

Data for this study were derived from the nationally representative 2018–2019 Cameroon Demographic and Health Survey. Analysis was done on 6080 married women in the reproductive age group (15–49 y) using Stata version 14 software. Pearson χ^2^ test and multilevel logistic regression analysis were conducted to examine the individual/household and community-level predictors of modern contraceptive use. Descriptive results were presented using frequencies and bar charts. Inferential results were presented using adjusted odds ratios (aORs) with 95% confidence intervals (CIs).

**Results:**

The results show only 18.3% (95% CI 16.8 to 19.8) of married women in Cameroon use modern contraceptives. Women's age (45–49 y; aOR 0.22 [95% CI 0.12 to 0.39]), education level (secondary education; aOR 2.93 [95% CI 1.90 to 4.50]), occupation (skilled manual; aOR 1.46 [95% CI 1.01 to 2.11]), religion (Muslim; aOR 0.63 [95% CI 0.47 to 0.84]), wealth quintile (richest; aOR 2.22 [95% CI 1.35 to 3.64]) and parity (≥5; aOR 3.59 [95% CI 2.61 to 4.94]) were significant individual/household-level predictors. Region (East; aOR 3.63 [95% CI 1.97 to 6.68]) was identified as a community-level predictor.

**Conclusions:**

Modern contraceptive use among married women in Cameroon is low. Women's education and employment opportunities should be prioritized, as well as interventions for married women, ensuring equity in the utilization of modern contraceptives across regions.

## Introduction

In the 21st century, family planning is considered an essential intervention for significant improvement of maternal and child health.^1–3^ As a result, ensuring universal coverage and utilization of modern contraceptives can help meet the Sustainable Development Goal (SDG) target of reducing maternal and neonatal morbidity and mortality.^3^ Furthermore, unwanted pregnancy, unsafe abortion-related social, mental and obstetric complications and maternal mortality can be averted through effective utilization of modern contraceptives.^4–7^ Also, modern contraceptives have individual benefits such as preventing unwanted pregnancy and its related emotional, financial and social problems such as discrimination by friends, family and the community.^8^ They also empower women by allowing continued education and the opportunity to work,^8^ positively contributing to societal and national development through increased women's participation in the labour market and optimization of limited resources due to reduced population growth.^9,10^

Globally, of the 1.1 billion women who needed family planning in 2019, 842 million used contraceptive methods, while the remaining 270 million had unmet needs.^8^ Worldwide, 75% of women are satisfied with their family planning needs, however, coverage is <50% in Central and West Africa.^8^ Although Cameroon has ratified the Family Planning 2020 (FP2020) initiative and committed to meeting the SDG target to reduce maternal and neonatal mortality through improved utilization of modern contraceptives,^3^ national family planning coverage remains extremely low.^11^ For example, according to the 2018 Cameroon Demographic and Health Survey (CDHS), the use of modern contraceptive methods among married women was <15%, although it was higher among sexually active non-married women, at 43%.^11^ In Cameroon, an increase in the utilization of modern contraceptive methods has not been satisfactory, from 4% to 15% from 1991 to 2018.^11^ Recently a community-based study in the northwest region estimated a modern contraceptive utilization rate of 13%.^4^

Several scholars in Cameroon^4,12,13^ and other African countries^14–20^ have shown that modern contraceptive use is linked to socio-economic conditions, women’s empowerment, partner support and geographic-related factors. The fact that few studies are available in Cameroon^4,12,13^ on modern contraceptive methods and continued poor uptake, especially among married women, motivated us to investigate wide-ranging predictors for modern contraceptive use in the country, using nationally representative data and a robust methodological approach. Therefore, this study examined individual/household and community-level predictors of modern contraceptive use among married women in Cameroon.

## Methods

### Data source

We extracted nationally representative data from the 2018–2019 Cameroon Demographic and Health Survey (CDHS) for analysis.^11^ The 2018–2019 CDHS collected data to monitor demographic and numerous health indicators, including modern contraceptive use. The National Institute of Statistics (NIS), in collaboration with several national and international organizations, completed the initiative with financial and technical support from United States Aid for International Development (USAID) and the Inner-City Fund (ICF) International, respectively.^11^ The survey applied a two-stage stratified cluster sampling technique. The probability proportional to size (PPS) technique was used to select large geographic locations, known as enumeration areas (EAs), from the sampling frame prepared in the recent population census (2005). In the second stage, a sample of households was selected using a systematic sampling technique of the EAs selected in the first stage.^11^ A total of 13 527 women ages 15–49 y and 6978 men ages 15–64 y were interviewed.^11^ The sample size for the study excluded pregnant and infecund married women and was limited to 6080 married women in the reproductive age group (15–49 y).^21,22^

### Study variables

#### Outcome variable

The main outcome variable of this study was modern contraceptive use. Married women who said they use one of the following methods were considered as modern contraceptive users: female sterilization (tubal ligation, laparotomy, voluntary surgical contraception for women), male sterilization (vasectomy, voluntary surgical contraception for men), contraceptive pills (oral contraceptives), intrauterine contraceptive device (IUD), injectables (Depo-Provera), implants (Norplant), female condom, male condom (prophylactic, rubber), diaphragm, contraceptive foam or jelly, lactational amenorrhea method (LAM), standard days method (SDM), country-specific modern methods and respondent-mentioned other modern contraceptive methods (including cervical cap, contraceptive sponge and others), but does not include abortions and menstrual regulation.^23–27^ Married women who used any method other than those mentioned above were not considered as modern contraceptive users. Other methods include periodic abstinence (rhythm, calendar method), withdrawal (coitus interruptus) and country-specific traditional methods and folk methods (locally described methods and spiritual methods of unproven effectiveness, such as herbs, amulets, gris-gris, etc.).^23–27^ The dichotomous outcome variable was coded as ‘yes’ if the married women used at least one of the above aforementioned modern contraceptive methods and ‘no’ if the women used none of the modern contraceptive methods.

#### Explanatory variables

Individual/household and community-level predictors were selected based on their proven significant association in prior literature.^14–20,23–27^

#### Individual/household-level predictors

Several individual/household-level predictors were included.^15–49^ We used the women's age, religion (Catholic, Protestant, other Christian, Muslim, other), ideal number of children (0–3, 4–5, ≥6), parity (0–2, 3–4, ≥5), media exposure and decision making. Media exposure was coded as ‘yes’ if the married woman had exposure for either of the three media sources (newspaper, radio, television) for at least once a week and ‘no’ otherwise. Women who made decisions alone or together with husbands on all three decision-making parameters (their own health, to purchase large household expenses, to visit family or relatives) were coded as ‘1’, otherwise they were coded as ‘0’.^28,29^ We also included the education level of the women and their husbands (no formal education, primary school, secondary school, higher), the women's occupation (not working, sales, agricultural self-employed, others) and the husband's occupation (not working, professional or technical or manager or clerical, sales, agricultural self-employed, services, skilled manual, unskilled manual). The wealth index was used as a proxy for economic status. Categorized as poorest, poor, middle, rich and richest, the DHS classifies the wealth index using household assets and ownership using principal component analysis (PCA), as explained elsewhere.^30^

#### Community-level predictors

Community-level predictors included place of residence (urban, rural), region (Adamawa, Centre [without Yaounde], Douala, East, Far North, Littoral [without Douala], North, Northwest, West, South, Southwest, Yaoundé), distance to a health facility (big problem, not a big problem), community socio-economic status (SES; low, medium, high) and community-level modern contraceptive knowledge (low, medium, high). The occupation, education and wealth of survey participants in each community were used to compute community-level SES. PCA was used to calculate women who were unemployed, uneducated and poor. A standardized score was derived, with a mean score (0) and standard deviation (1). These were then categorized into tertile 1 (lowest score, least disadvantaged and greater SES), tertile 2 and tertile 3 (highest score, most disadvantaged and lowest SES). To determine the community literacy level, respondents who attended higher than secondary school were assumed to be literate, while all other respondents were given a sentence to read and were considered literate if they could read all or part of the sentence. Therefore, high literacy included respondents who had higher than a secondary education or had no school/primary/secondary education but could read a whole sentence. Medium literacy was respondents without school/primary/secondary education who could read part of the sentence. Low literacy was respondents who had no school/primary/secondary education and could not read at all. These were categorized into appropriate tertiles, where tertile 1 (lowest score, least disadvantaged) was high community literacy, tertile two (medium score) was medium community literacy and tertile 3 (highest score, most disadvantaged) was low community literacy.

#### Statistical analysis

Using Stata version 14 software (StataCorp, College Station, TX, USA), analyses were conducted using the following steps. First, frequencies and percentages, including the prevalence of modern contraceptive use, were used to present the demographic characteristics of the respondents. Next, bivariate analysis (Pearson χ^2^ test) determined whether there was an association between each individual/household and community-level variables and modern contraceptive use and p-values <0.05 were used as a cut-off point. For all variables that had significant associations in the χ^2^ test, a multicollinearity test was performed using the variance inflation factor (VIF) to test the presence or absence of collinearity, and the result showed no evidence of multicollinearity (mean VIF 1.76, minimum VIF 1.03, maximum VIF 3.59). Two-level and multilevel logistic regression analyses were then carried out for all independent variables that had significant associations in the χ^2^ test. Using four steps, we constructed four models. First, we constructed the empty/null model, which represents the model that focuses on the variance in the outcome variable (modern contraceptive use), accredited to the clustering at the primary sampling units (PSUs) (model 0). Second, the individual/household-level factors were included in a model to assess their association with modern contraceptive use (model 1). Third, we developed a model that included only the community-level variables, to assess their association with modern contraceptive use (model 2). We used the term community to describe clustering within the same geographical living environment. Communities were based on sharing a common PSU within the DHS data. Finally, all the variables were included for a complete model (model 3). The multilevel logistic regression model yielded fixed and random effects.^26,27,31–33^ Reported as adjusted odds ratios (aORs) with their 95% confidence intervals (CIs), the fixed effects (measures of association) revealed the association between the independent variables and the dependent/outcome variable. Intracluster correlation assesses the random effects (measures of variations).^34^ The likelihood ratio (LR) checks for model adequacy, while Akaike's information criterion (AIC) was used to measure how well the different models best fit the data. We also reported the intraclass correlation coefficient (ICC), ρ, for each model. The ICC is the proportion of variance in the outcome variable (modern contraceptive use) that is explained by the grouping structure of the hierarchical model. It is calculated as a ratio of group-level error variance over the total error variance:

$$\begin{equation*}\rho = \frac{{\sigma _{{u_0}}^2}}{{\sigma _{{u_0}}^2 + \sigma _e^2}}\end{equation*}$$

where $\sigma _{{u_0}}^2$ is the variance of the level 2 residuals and $\sigma _e^2 $ is the variance of the level 1 residuals. In other words, the ICC reports the amount of variation unexplained by any predictors in the model that can be attributed to the grouping variable as compared with the overall unexplained variance (within and between variance). The complex structure and design of the CDHS data were considered using the svyset command module in Stata; all three design elements (weight, cluster and strata) were considered. For the preparation of this article, we followed the Strengthening the Reporting of Observational Studies in Epidemiology guidelines.^35^

#### Ethical consideration

Data for this study were obtained from a secondary dataset with de-identified information. To have access to the data, the authors obtained and were granted approval to use the dataset by MEASURE DHS. The data are secondary and available in the public domain, therefore no further approvals were required for this study. Details regarding data and ethical standards are available at http://goo.gl/ny8T6X.

## Results

### Sociodemographic characteristics of respondents

As shown in Table 1, 7.4% were adolescents (15–19 y of age) and 51.4% of the respondents were rural residents. A total of 28.5% and 25.9% of participants had not attended formal education and were not working, respectively. A total of 42.7% of the participants were not exposed to media (newspaper, radio or television) at least once a week. Regarding decision making, only 46.6% of married women had decided, either alone or with their husband, on all three of the decision-making parameters—their own health, to purchase large household expenses and to visit family or relatives.

**Table 1.** Frequency distribution of respondents and modern contraceptive use distribution across explanatory variables among married women: evidence from the 2018–2019 CDHS

		Modern contraceptive (weighted %)		
Variable	Number (weighted %)	No	Yes	χ^2^, p-value
Overall prevalence	6080 (18.3)			
Age (years)				65.35, <0.001
15–19	465 (7.39)	87.85	12.15	
20–24	977 (15.77)	78.32	21.68	
25–29	1325 (22.29)	79.36	20.64	
30–34	1221 (20.78)	78.73	21.27	
35–39	976 (16.08)	82.14	17.86	
40–44	691 (10.86)	87.42	12.58	
45–49	425 (6.83)	90.08	9.92	
Women's education level				384.84, <0.001
No formal education	1506 (28.51)	96.23	3.77	
Primary school	1971 (30.19)	80.38	19.62	
Secondary school	2274 (35.66)	73.23	26.77	
Higher	329 (5.64)	69.71	30.29	
Husband's education level				318.80, <0.001
No formal education	1211 (23.76)	96.32	3.68	
Primary school	1865 (30.89)	81.44	18.56	
Secondary school	2268 (36.03)	76.12	23.88	
Higher	546 (9.32)	67.55	32.45	
Women's occupation				138.66, <0.001
Not working	1664 (25.85)	85.06	14.94	
Sales	1219 (19.87)	78.22	21.78	
Agricultural	2051 (34.93)	86.86	13.14	
Services	635 (10.43)	71.16	28.84	
Skilled manual	325 (5.51)	71.79	28.21	
Other	186 (3.42)	73.29	26.71	
Husband's occupation				181.20, <0.001
Not working	173 (2.53)	88.52	11.48	
Professional or technical or manager or clerical or clerical	374 (5.83)	74.38	25.62	
Sales	1035 (16.46)	79.01	20.99	
Agricultural self-employed	2343 (39.92)	89.02	10.98	
Services	709 (11.26)	70.49	29.51	
Skilled manual	1219 (20.38)	77.60	22.40	
Unskilled manual	202 (3.63)	79.35	20.65	
Religion				173.08, <0.001
Catholic	2094 (34.83)	75.23	24.77	
Protestant	1613 (24.11)	80.17	19.83	
Muslim	1706 (30.21)	91.16	8.84	
Others	667 (10.84)	79.96	20.04	
Wealth status				254.65, <0.001
Poorest	1067 (21.26)	94.91	5.09	
Poorer	1249 (19.69)	84.26	15.74	
Middle	1407 (19.50)	79.69	20.31	
Richer	1236 (19.60)	76.94	23.06	
Richest	1121 (19.96)	71.97	28.03	
Media exposure				237.22, <0.001
No	2456 (42.74)	90.58	9.42	
Yes	3624 (57.26)	75.15	24.85	
Decision making				63.52, <0.001
No	3069 (53.37)	85.44	14.56	
Yes	3011 (46.63)	77.52	22.48	
Ideal number of children				183.94, <0.001
0–3	697 (11.19)	72.6	27.4	
4–5	2181 (33.81)	74.94	25.06	
≥6	3202 (55.00)	87.79	12.21	
Parity				29.95, <0.001
0–2	1221 (19.56)	86.17	13.83	
3–4	2785 (45.75)	79.11	20.89	
≥5	2074 (34.69)	82.73	17.27	
Place of residence				135.67, <0.001
Urban	2929 (48.65)	75.82	24.18	
Rural	3151 (51.35)	87.36	12.64	
Distance to health facility				28.45, <0.001
Not a big problem	2580 (42.10)	84.84	15.16	
Big problem	3500 (57.90)	79.49	20.51	
Region				353.84, <0.001
Adamawa	541 (5.51)	92.88	7.12	
Centre (without Yaounde)	587 (8.89)	72.83	27.17	
Douala	458 (11.03)	79.58	20.42	
East	510 (5.85)	64.48	35.52	
Far North	790 (18.21)	91.90	8.10	
Littoral (without Douala)	384 (3.53)	81.25	18.75	
North	775 (15.67)	91.64	8.36	
Northwest	305 (5.96)	75.36	24.64	
West	578 (10.18)	79.02	20.98	
South	559 (4.50)	82.50	17.50	
Southwest	127 (1.56)	76.81	23.19	
Yaounde	466 (9.12)	67.97	32.03	
Community literacy level				236.85, <0.001
Low	2492 (44.66)	90.10	9.90	
Medium	1879 (26.66)	76.91	23.09	
High	1709 (28.68)	73.24	26.76	
Community SES				193.01, <0.001
Low	2935 (49.18)	88.71	11.29	
Medium	1319 (18.54)	76.14	23.86	
High	1826 (32.28)	74.35	25.65	
Community-level modern contraceptive knowledge				75.51, <0.001
Low	2128 (31.65)	87.46	12.54	
Medium	1978 (33.38)	81.38	18.62	
High	1974 (34.97)	76.92	23.08	

### Overall prevalence of modern contraceptive use

**Figure 1.**
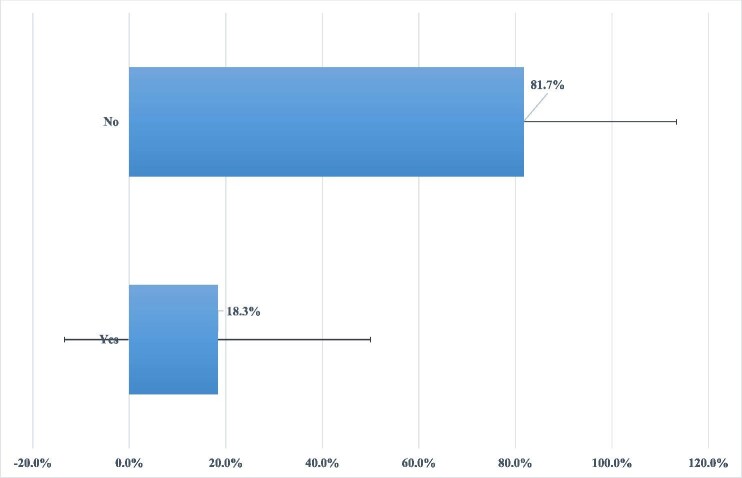
Prevalence of modern contraceptive use among married women in Cameroon: evidence from 2018–2019 CDHS.

The prevalence of modern contraceptive use among married women was 18.3% (95% CI 16.8 to 19.8) (Figure 1).

### Distribution of modern contraceptive use across explanatory variables

Table 1 shows the distribution of modern contraceptive use across explanatory variables and subgroups. For instance, only 9.9% of married women ages 45–49 y and 12.2% of married adolescents (15–19 y) used modern contraceptives, while the prevalence rose to 21.7% among married women ages 20–24 y. Modern contraceptive use among married women with no formal education was 3.8% and among those who attended higher education was 30.3%. A total of 3.7% of married women whose husbands had not attended formal education used modern contraceptives, compared with 32.5% of married women whose husbands had higher education. Modern contraceptive use ranged from 5.1% to 28.0% for married women in the poorest and richest households, respectively. The study showed that the prevalence of modern contraceptive use ranged from 14.6% to 22.5% among married women with decision-making power and those with no decision-making power, respectively (Table 1).

### Predictors of modern contraceptive use

#### Fixed effects (measures of associations) results

##### Individual/household-level predictors.

As shown in Table 2, several individual/household-level predictors were significantly associated with modern contraceptive use. We found lower odds of modern contraceptive use among married women ages 40–44 y (aOR 0.32 [95% CI 0.20 to 0.52]) and 45–49 y (aOR 0.22 [95% CI 0.12 to 0.39]) as compared with married adolescents (15–19 y). The study showed higher odds of modern contraceptive use among married women who had attended primary school (aOR 2.52 [95% CI 1.71–3.71]), secondary school (aOR 2.93 [95% CI 1.90 to 4.50]) and higher education (aOR 2.48 [95% CI 1.29 to 4.75]) as compared with married women who had not attended any formal education. Similarly, we found higher odds of modern contraceptive use among married women whose husbands attended primary school (aOR 1.96 [95% CI 1.24 to 3.09]), secondary school (aOR 1.80 [95% CI 1.15 to 2.83]) and higher (aOR 2.23 [95% CI 1.32 to 3.77]) as compared with married women whose husbands had not attended formal education. Women's occupation (skilled manual; aOR 1.46 [95% CI 1.01 to 2.11]) was a significant predictor for modern contraceptive use.

**Table 2.** Individual/household and community-level predictors for modern contraceptive use among married women: evidence from the 2018–2019 CDHS

Variables	Model 1	Model 2	Model 3
Age (years)			
15–19	Ref		Ref
20–24	1.01 (0.68 to 1.51)		1.05 (0.70 to 1.57)
25–29	0.82 (0.55 to 1.23)		0.87 (0.58 to 1.31)
30–34	0.69 (0.45 to 1.05)		0.74 (0.48 to 1.14)
35–39	0.49 (0.31 to 0.77)**		0.53 (0.33 to 0.84)**
40–44	0.29 (0.18 to 0.47)***		0.32 (0.20 to 0.52)***
45–49	0.20 (0.11 to 0.35)***		0.22 (0.12 to 0.39)***
Women's education level			
No formal education	Ref		Ref
Primary school	2.60 (1.77 to 3.81)***		2.52 (1.71 to 3.71)***
Secondary school	2.94 (1.92 to 4.50)***		2.93 (1.90 to 4.50)***
Higher	2.52 (1.32 to 4.81)**		2.48 (1.29 to 4.75)**
Husband's education level			
No formal education	Ref		Ref
Primary school	2.06 (1.30 to 3.25)**		1.96 (1.24 to 3.09)**
Secondary school	1.90 (1.21 to 3.00)**		1.80 (1.15 to 2.83)*
Higher	2.36 (1.39 to 4.01)**		2.23 (1.32 to 3.77)**
Women's occupation			
Not working	Ref		Ref
Sales	1.19 (0.93 to 1.51)		1.15 (0.90 to 1.46)
Agricultural	1.28 (0.96 to 1.69)		1.22 (0.90 to 1.64)
Services	1.28 (0.93 to 1.75)		1.22 (0.90 to 1.67)
Skilled manual	1.54 (1.07 to 2.22)*		1.46 (1.01 to 2.11)*
Other	1.51 (0.90 to 2.56)		1.48 (0.87 to 2.51)
Husband's occupation			
Not working	Ref		Ref
Professional or technical or manager or clerical or clerical	1.55 (0.73 to 3.27)		1.41 (0.67 to 2.97)
Sales	1.55 (0.75 to 3.19)		1.39 (0.68 to 2.85)
Agricultural self-employed	1.24 (0.62 to 2.50)		1.16 (0.58 to 2.33)
Services	1.90 (0.95 to 3.82)		1.67 (0.83 to 3.35)
Skilled manual	1.42 (0.69 to 2.90)		1.31 (0.64 to 2.66)
Unskilled manual	1.34 (0.67 to 2.68)		1.24 (0.62 to 2.48)
Religion			
Catholic	Ref		Ref
Protestant	0.86 (0.70 to 1.06)		0.92 (0.74 to 1.14)
Muslim	0.61 (0.46 to 0.81)**		0.63 (0.47 to 0.84)**
Other	0.96 (0.74 to 1.25)		0.93 (0.72 to 1.21)
Wealth status			
Poorest	Ref		Ref
Poorer	2.08 (1.47 to 2.93)***		2.09 (1.47 to 2.97)***
Middle	2.18 (1.48 to 3.20)***		2.08 (1.39 to 3.10)***
Richer	1.98 (1.32 to 2.96)**		1.82 (1.17 to 2.81)**
Richest	2.32 (1.49 to 3.60)***		2.22 (1.35 to 3.64)**
Media exposure			
No	Ref		Ref
Yes	1.25 (0.95 to 1.65)		1.25 (0.95 to 1.65)
Decision making			
No	Ref		Ref
Yes	1.05 (0.87 to 1.27)		1.03 (0.86 to 1.25)
Ideal number of children			
0–3	Ref		Ref
4–5	0.76 (0.58 to 0.99)*		0.74 (0.57 to 0.97)*
≥6	0.55 (0.40 to 0.74)***		0.54 (0.40 to 0.73)***
Parity			
0–2	Ref		Ref
3–4	2.11 (1.66 to 2.67)***		2.08 (1.64 to 2.63)***
≥5	3.69 (2.67 to 5.10)***		3.59 (2.61 to 4.94)***
Place of residence			
Urban		Ref	Ref
Rural		0.66 (0.48 to 0.91)*	0.77 (0.58 to 1.01)
Distance to health facility			
Not a big problem		Ref	Ref
Big problem		1.09 (0.88 to 1.34)	1.03 (0.82 to 1.29)
Region			
Adamawa		Ref	Ref
Centre (without Yaounde)		3.23 (1.76 to 5.92)***	1.63 (0.92 to 2.89)
Douala		1.60 (0.84 to 3.03)	0.94 (0.52 to 1.71)
East		6.89 (3.61 to 13.12)***	3.63 (1.97 to 6.68)***
Far north		1.25 (0.65 to 2.37)	1.46 (0.83 to 2.55)
Littoral (without Douala)		2.11 (1.11 to 4.03)*	1.19 (0.63 to 2.24)
North		1.33 (0.77 to 2.30)	1.56 (0.95 to 2.58)
Northwest		3.70 (1.96 to 6.96)***	1.65 (0.96 to 2.85)
West		2.34 (1.26 to 4.33)**	1.38 (0.78 to 2.46)
South		1.85 (0.93 to 3.67)	1.05 (0.55 to 1.99)
Southwest		1.86 (0.88 to 3.91)	1.17 (0.55 to 2.47)
Yaounde		2.88 (1.52 to 5.46)**	1.71 (0.94 to 3.11)
Community literacy level			
Low		Ref	Ref
Medium		1.67 (1.18 to 2.37)**	0.99 (0.73 to 1.35)
High		1.59 (1.06 to 2.39)*	0.92 (0.63 to 1.34)
Community SES			
Low			
Medium		1.35 (0.98 to 1.87)	1.19 (0.88 to 1.61)
High		1.19 (0.83 to 1.71)	1.07 (0.75 to 1.52)
Community-level modern contraceptive knowledge			
Low			
Medium		1.32 (1.01 to 1.73)*	1.27 (0.98 to 1.64)
High		1.33 (1.01 to 1.75)*	1.15 (0.88 to 1.51)

Values presented as aOR (95% CI).

Model 1: included only individual/household-level predictors; model 2: included only community-level predictors; model 3: included both individual/household and community-level predictors. Significant at ***p<0.001, **p<0.01, *p<0.05. Ref: reference. Other occupation included clerical, services, skilled and unskilled.

In this study we found lower odds of modern contraceptive use among Muslim married women (aOR 0.63 [95% CI 0.47 to 0.84]) compared with Catholic married women. Moreover, there were higher odds of modern contraceptive use among married women from poor (aOR 2.09 [95% CI 1.47 to 2.97]), middle (aOR 2.08 [95% CI 1.39 to 3.10]), rich (aOR 1.82 [95% CI 1.17 to 2.81]) and richest (aOR 2.22 [95% CI 1.35 to 3.64]) households as compared with married women in the poorest quintile. Married women with an ideal number of children of 4–5 (aOR 0.74 [95% CI 0.57 to 0.97]) and ≥6 (aOR 0.54 [95% CI 0.40 to 0.73]) had lower odds of modern contraceptive use than married women with an ideal number of 0–3 children. Furthermore, the study showed higher odds of modern contraceptive use among married women with a parity history of 3–4 (aOR 2.08 [95% CI 1.64 to 2.63]) and ≥5 (aOR 3.59 95% CI 2.61 to 4.94]) as compared with married women with a parity history of 0–2.

##### Community-level predictors.

As shown in Table 2, we found that region and community-level modern contraceptive knowledge were significant community-level predictors for modern contraceptive use among married women in Cameroon. More specifically, the study showed higher odds of modern contraceptive use among married women who were living in the East region (aOR 3.63 [95% CI 1.97 to 6.68]) as compared with married women who lived in the Adamawa region.

#### Random effects (measures of variations) results

As shown in Table 3, the empty model (model 0) shows significant variations in the prevalence of modern contraceptive use across the clusters (σ^2^=0.93 [95% CI 0.72 to 1.20]). The empty model further shows that 18% of the total variance in the prevalence of modern contraceptive use was attributed to between-cluster variations (ICC 0.20). The between-cluster variations decreased from 20% in model 0 to 8% in model 1 (individual/household only model), then remained constant as 8% in model 2 (community-level model only) and finally the ICC decreased to 5% in model 3 (complete model). This indicates that differences in the clusters of PSUs account for variations in modern contraceptive use. The best-fit model (model 3) was determined using the highest log-likelihood (−2442.02) and lowest AIC (4998.05) (Table 3).

**Table 3.** Random effect results for individual/household and community-level predictors for modern contraceptive use among married women: evidence from the 2018–2019 CDHS

Random effects result	Model 0 (empty model)	Model 1	Model 2	Model 3
PSU variance (95% CI)	0.93 (0.72 to 1.20)	0.34 (0.22 to 0.52)	0.36 (0.24 to 0.52)	0.22 (0.13 to 0.37)
ICC	0.20	0.08	0.08	0.05
LR test	269.33	53.97	57.74	21.61
Wald χ^2^, p-value	Ref	387.37, <0.001	254.48, <0.001	522.39, <0.001
Model fitness				
Log-likelihood	−2821.65	−2471.93	−2719.09	−2442.02
AIC	5647.32	5019.864	5480.193	4998.05
PSU	428	428	428	428

Ref: reference.

## Discussion

Achieving the third SDG of reducing maternal morbidity and mortality and achieving universal health coverage to include access to essential healthcare services by 2030 have been issues of great concern in developing countries.^36,37^ Two-thirds of the global maternal deaths (196 000) take place in sub-Saharan Africa (SSA).^38^ Maternal, neonatal and infant morbidity and mortality can be averted using modern contraceptives.^9,39,40^ Only 18.3% of married women use modern contraception in Cameroon, which has a high maternal mortality ratio.^41,42^ Using recent nationally representative data, we examined a broader range of individual/household and community-level predictors for modern contraceptive use among married women in Cameroon. We found that women's age was associated with modern contraceptive use with lower odds among older women compared with younger married women, like prior studies in Senegal,^26^ Ethiopia^43^ and Uganda.^44^ Possible explanations include higher education levels among the younger women^45^ and an increased likelihood of positive communication between younger women and their partner/husband about modern contraceptives.^26,46^

Consistent with previous studies in Ghana^47^ and Uganda,^48^ we found that women's education level affects modern contraceptive utilization. Education can promote decision-making capacity and autonomy, increase choices for economic freedom and affect women's current and future fertility plans.^49^ Furthermore, increased contraceptive uptake and fertility control were seen in communities where women achieved higher levels of education.^50^

We also found that the husband's education level had an influence on modern contraceptive utilization, as documented in previous studies in Senegal^19^ and SSA.^15^ It is possible that educated husbands are more likely to use modern contraceptives, which can contribute to fertility control, especially in families where men are the sole decision maker about fertility.^16^ Additionally, this might be due to better communication between spouses and approval of the husband.^50,51^ Better communication and discussion about family planning augment understanding and facilitate agreement on contraceptive issues between couples, which potentially leads to better uptake of modern contraceptive use.^53,54^

Like previous studies in Bangladesh^55^ and India,^56^ we identified that women who were employed had better utilization of modern contraceptives than non-employed women. Moreover, the study showed higher odds of modern contraceptive use among wealthier women than poor, as reported in previous studies in Senegal,^26^ Mali,^27^ Burkina Faso,^57^ Malawi^58^ and Bangladesh.^59^ This may be due to women from wealthier households having a higher SES that enables them to have better access to media and healthcare services.^26,27,60,61^

Moreover, religion had a significant association with modern contraceptive use, consistent with a previous study in Bangladesh.^56^ Religion plays a vital role in the lives of many people, with 88.3% of the global population relating with faith.^62^ As a result, faith leaders are a significant and often powerful factor in the lives of their followers.^62^ Also, many faith leaders have the skills and the platform to speak out and deliver key messages to their congregations.^62^ So working with faith leaders offers an opportunity to reach many people with messages distributed by those who are already significantly valued within their societies.^62^

Comparable with previous studies in Mali,^27^ Bangladesh^63^ and Indonesia,^64^ women who planned to have more children or those who perceived and desired to have more children were less likely to use modern contraceptives.^65^ The plausible reason for not using contraception among married women with a higher ideal number of children might be due to children being considered a prized resource for future household-level economic growth. This may be beneficial in resource-limited countries such as Cameroon; having more children potentially leads to additional household income due to the increased likelihood of more household members participating in the labour force.^59,66^ On the other hand, better utilization of modern contraceptives is reported among women who do not want to have more children, usually non-married women.^27,67^

Furthermore, we found that parity was a significant individual-level predictor for modern contraceptive use, with better use among married women of higher parity, as reported in prior studies in Uganda.^44,45^ This might be to limit future pregnancies.^44,45^ Comparable with prior studies in Bangladesh^27,29^ and Senegal,^17^ we found that modern contraceptive utilization significantly varied across regions. The plausible reason might be related to the difference in family planning services and the number of health facilities across regions.^17,68^ Furthermore, uneven distribution of health structures and health personnel across regions might make a difference in contraceptive use across regions.^69^ Variations in contraceptive supplies is another reason^70^ that needs further supply-related evaluation studies.^4^

### Strength and limitations

Investigating wide-ranging predictors of modern contraceptive use using a multilevel modelling approach and nationally representative data are the main strengths of this study. The main limitation of this study is the use of cross-sectional data, which makes it impossible to infer causality from the associations observed in this study. Moreover, since the data were self-reported, the probabilities of recall and reporting biases should be considered. Predictors of modern contraceptive use were wide-ranging and multidimensional, but the choice of the independent variables was restricted to existing data from the CDHS. This study could not account for several residual confounders (i.e., cultural barriers). Another limitation is social desirability bias, since the DHS questionnaires are interviewer administered.

## Conclusions

The findings show modern contraceptive use among married women in Cameroon is low. Women's age, education level, occupation, religion, wealth quintile, ideal number of children and parity, as well as the husband's education level, were significant individual/household-level predictors, whereas region was a community-level predictor. Improving women's employment opportunities through education and skilled occupations will ensure equity in modern contraceptive use across regions.

## Data Availability

The datasets for this study were sourced and analysed from the DHS and are available from: http://dhsprogram.com/data/available-datasets.cfm.
